# Temperature and Vibration Dependence of the Faraday Effect of Gd_2_O_3_ NPs-Doped Alumino-Silicate Glass Optical Fiber

**DOI:** 10.3390/s18040988

**Published:** 2018-03-27

**Authors:** Seongmin Ju, Jihun Kim, Kadathala Linganna, Pramod R. Watekar, Seong Gu Kang, Bok Hyeon Kim, Seongjae Boo, Youjin Lee, Yong Ho An, Cheol Jin Kim, Won-Taek Han

**Affiliations:** 1 School of Electrical Engineering and Computer Science/Advanced Photonics Research Institute, Gwangju Institute of Science and Technology, Gwangju 61005, Korea; jusm@gist.ac.kr (S.J.); bbashya1012@gist.ac.kr (J.K.); prwatekar@gmail.com (P.R.W.); kseonggu@gist.ac.kr (S.G.K.); bhkim@gist.ac.kr (B.H.K.); 2 Advanced Optical Lens Research Center, Korea Photonics Technology Institute, Gwangju 61007, Korea; lingannasvu@gmail.com; 3 Solar City Center, Korea Institute of Industrial Technology, Gwangju 61012, Korea; sboo@kitech.re.kr; 4 Power System Laboratory, Korea Electric Power Corporation Research Institute, Daejeon 34056, Korea; youjin.lee@kepco.co.kr (Y.L.), yonghoan@kepco.co.kr (Y.H.A.); 5 Department of Nano and Advanced Materials Engineering, Gyeongsang National University, Jinju 52828, Korea; cjk@gnu.ac.kr

**Keywords:** faraday effect, nano-particles, optical fiber, optical absorption, temperature dependence

## Abstract

All-optical fiber magnetic field sensor based on the Gd_2_O_3_ nano-particles (NPs)-doped alumino-silicate glass optical fiber was developed, and its temperature and vibration dependence on the Faraday Effect were investigated. Uniformly embedded Gd_2_O_3_ NPs were identified to form in the core of the fiber, and the measured absorption peaks of the fiber appearing at 377 nm, 443 nm, and 551 nm were attributed to the Gd_2_O_3_ NPs incorporated in the fiber core. The Faraday rotation angle (FRA) of the linearly polarized light was measured at 650 nm with the induced magnetic field by the solenoid. The Faraday rotation angle was found to increase linearly with the magnetic field, and it was about 18.16° ± 0.048° at 0.142 Tesla (T) at temperatures of 25 °C–120 °C, by which the estimated Verdet constant was 3.19 rad/(T∙m) ± 0.01 rad/(T∙m). The variation of the FRA with time at 0.142 T and 120 °C was negligibly small (−9.78 × 10^−4^ °/min). The variation of the FRA under the mechanical vibration with the acceleration below 10 g and the frequency above 50 Hz was within 0.5°.

## 1. Introduction

Lanthanide ions have been studied extensively owing to their unique response to various parameters such as magnetic field, optical interference, and biomedical stimuli [1,2,3,4,5,6,7]. Most of the lanthanide materials show strong magneto-optic interactions attributed to their 4*f* electrons [4,5,8,9,10,11,12,13,14,15]. Especially, the well-known Faraday rotation under magnetic field involves the transition 4*f*^n^ to 4*f*^n−1^5*d* of lanthanide ions [8,9,10,11,12,13,14,15]. Especially, glasses that contain lanthanide ions are suitable as strong magneto-optic media due to lower absorption in the spectral regions of interest, and their durability is an added advantage [5]. Furthermore, fiber optic devices using optical fiber incorporated with lanthanide ions are useful for all-optical applications and offer advantages of miniaturization, high sensitivity, immunity to electromagnetic interference, real-time remote sensing, and cost effectiveness. Earlier, therefore, the magneto-optic properties, in particular the Verdet constants of silicate glass optical fibers doped with lanthanide ions such as Tb^3+^ ions (−32.1 rad/(T·m) at 1053 nm) and Eu^3+^ ions (−4.6 rad/(T·m) at 660 nm), have been extensively studied [13,14]. Our group has also reported the Verdet constants of silicate glass optical fibers incorporated with Eu^2+^ ions (2.6 rad/(T·m) at 633 nm), as well as Cu (2.1 rad/(T·m) at 660 nm), CdSe (7.2 rad/(T·m) at 633 nm), and CdMnTe (5.1 rad/(T·m) at 660 nm) [15,16,17,18,19].

Among the lanthanides-doped optical fibers, optical fibers doped with paramagnetic dopants such as Ce^3+^, Pr^3+^, Nd^3+^, Eu^3+^, Tb^3+^, and Dy^3+^ exhibited enhanced magneto-optic properties compared to the fibers doped with diamagnetic materials such as Sm^3+^, Gd^3+^, and Yb^3+^. However, the paramagnetic optical fibers have larger temperature sensitivity than the diamagnetic optical fibers. This is due to the difference in the magnetization mechanisms, such that atoms possess magnetic dipole moments at the microscopic level in paramagnetic material but not in diamagnetic material. Thus, optical fibers doped with diamagnetic materials are suitable as a magneto-optic component, with its temperature insensitivity and compact size. Field applications in magneto-optic sensors often require high accuracy over a wide temperature range. In addition, mechanical perturbations should not disturb the signal in order to avoid erroneous triggering of the magneto-optic sensor system [20]. Therefore, magneto-optic devices such as microwave filters, optical isolators, and optical current sensors that use the optical fibers doped with diamagnetic materials are very attractive, because no additional temperature and vibration compensation systems are needed. Among diamagnetic lanthanides, nano-particles (NPs) of gadolinium oxide (Gd_2_O_3_) are of great interest due to the linear response of the Faraday effect, their relative stability with respect to temperature, and the large atomic magnetic momentum from seven unpaired electrons of the trivalent gadolinium ion Gd^3+^ [4,5,9,10,11,12].

Optical fibers doped with Gd_2_O_3_ NPs are then expected to be an attractive material for magneto-optic devices, which require excellent optical transparency, low optical loss, and high durability [5,10]. However, since magneto-optic sensitivity is known to decrease with increasing wavelength, optical fibers with short cutoff wavelengths near visible wavelength are needed for better performance. We have fabricated the Gd_2_O_3_ NPs-doped specialty optical fiber that is in single mode at 650 nm by using the modified chemical vapor deposition (MCVD) and the drawing process. We present the fabrication and the experimental results related to the Faraday effect of the alumino-silicate glass optical fiber incorporated with Gd_2_O_3_ NPs. Especially, the temperature and vibration dependence are discussed on Faraday rotation angle (FRA) under magnetic field induced by the DC solenoid.

## 2. Theory

### Temperature Dependence of Faraday Rotation in Magnetic Materials

Although the glass optical fibers doped with diamagnetic materials exhibit weaker magneto-optic properties than the fibers doped with paramagnetic materials, these fibers have small temperature sensitivity. In paramagnetic materials, which have microscopic magnetization in the absence of an applied magnetic field, some or all of the individual atoms possess magnetic dipole moments that are randomly aligned. However, in diamagnetic materials, no microscopic or macroscopic magnetization occurs in the absence of an applied magnetic field. Therefore, Faraday effect in diamagnetic materials arises from the splitting of the upper level of an optical transition. Additionally, the temperature-dependent Verdet constant of diamagnetic materials, *V_dia_*, is well represented by the expression as follows [1,8,21,22]:

(1) $$\frac{dV_{dia}}{dT}=\frac{e\lambda }{2mc}\left[\frac{d}{d\lambda }\left(\frac{dn}{dT}\right)\right]$$

in which *e* is the electronic charge, *λ* is the wavelength, *m* is the electronic mass, *c* is the speed of light, *n* is the refractive index, and *T* is temperature at the measurement. The variation of the Verdet constant with temperature in diamagnetic materials is only related to the temperature dependence of refractive index from Equation (1). 

In the case of the paramagnetic materials, on the other hand, the Verdet constant, *V_par_*, can be expressed by following expression using quantum mechanics [1,23,24,25]:

(2) $$V_{par}=\frac{4\pi^{2}\nu^{2}\chi_{par}}{g\beta ch}\Sigma_{a,b}\left(\frac{C_{ab}}{\nu^{2}-\nu_{ab}^{2}}\right)$$

in which *ν* is the frequency of the incident light, *g* is the Landé splitting factor, *β* is the Bohr magneton number, *c* is the speed of the light, *h* is the Planck`s constant, *C_ab_* is a transition probability, and *ν_ab_* is the frequency associated with the transition. Here, the paramagnetic susceptibility *χ_par_* is given by [1,8]

(3) $$\chi_{par}=\frac{Ng^{2}\left[J\left(J+1\right)\right]\mu_{B}^{2}}{3kT}$$

in which *N* is the number of atoms or ions per unit volume, *J* is the spin orbit quantum number, *k* is Boltzmann’s constant, *μ_B_* is the Bohr magneton, and *T* is temperature at the measurement. Since the Verdet constant in paramagnetic materials is inversely proportional to temperature from the Equations (2) and (3), the variation of the Verdet constant with temperature in paramagnetic materials is primarily due to the temperature dependence of paramagnetic susceptibility.

## 3. Experiments

### 3.1. Preform Fabrication

An alumino-silicate glass optical fiber preform incorporated with Gd_2_O_3_ NPs in core region was fabricated by the MCVD process with the modified solution doping process. For a core part of the preform, porous silica layers were deposited onto the inner surface of a silica glass tube at temperature of about 1650 °C by the MCVD process using silicon tetrachloride (SiCl_4_) as a precursor. The doping was carried out by soaking the deposited porous silica glass layers with a solution containing Al and Gd prepared by dissolving 0.25 mole and 0.05 mole of reagent grade AlCl_3_·6H_2_O powder (High Purity Chemicals Co., Ltd., Sakado, Japan, 99.9%) and Gd_2_O_3_ powder (Cerac Inc., Milwaukee, WI, USA, 99.9%) in hydrochloric acid solution (Daejung Co., Ltd., Siheung-si, Korea, 35%), respectively. Note that Al was added instead of germanium(Ge) to increase the refractive index of the preform core. Moreover, the formation of Gd_2_O_3_ NPs at higher concentration is expected to be facilitated by addition of Al [26,27,28,29]. The soaked silica glass layers were dried to evaporate the residual solvent and only solutes (Al and Gd) remaining in the deposited porous layers. Then, the doped porous silica layers were sintered, and the tube was collapsed and sealed into a preform. 

### 3.2. Verification of Existence of Gd_2_O_3_ Nano-Particles

To confirm the formation of Gd_2_O_3_ NPs in the core of the fiber preform, the core was examined by the transmission electron microscopy (TEM; Tecnai, Hillsboro, OR, USA, G2 S-Twin 300 KeV), the X-ray photoelectron spectrometer (XPS; Thermo Scientific™, Waltham, MA, USA, K-Alpha™), and the UV-VIS spectrophotometer (Varian, Palo Alto, CA, USA, Cary500Scan). Also, to verify the existence of Gd_2_O_3_ NPs in the core of the fiber, microstructural measurements were conducted using the Cs-corrected scanning transmission electron microscopy (STEM; JEOL, Peabody, MA, USA, JEM-ARM200F) with energy dispersive X-ray spectroscopy (EDS). The elemental compositions were determined by the STEM analysis. The results are discussed in the next section.

### 3.3. Optical Fiber Drawing and Characterization

**Figure 1:**
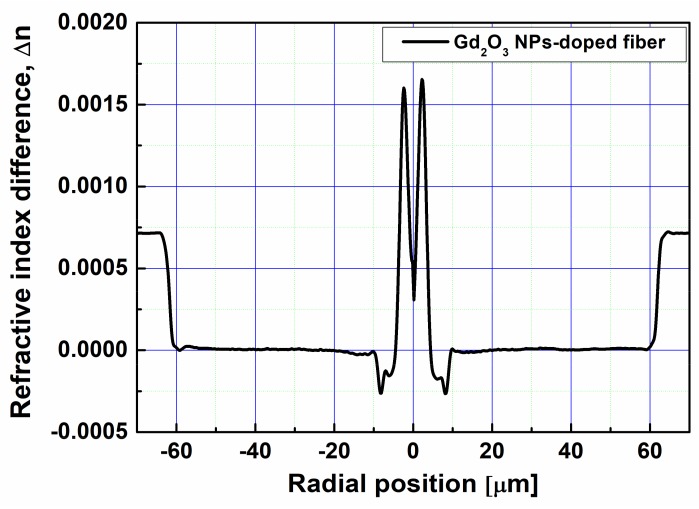
Refractive index profile of the Gd_2_O_3_ NPs-doped fiber.

The preform was drawn into an optical fiber using the drawing tower at 2100 °C. The refractive index profile of the optical fiber was measured by using the optical fiber analyzer (Interfiber Analysis, Sharon, MA, USA, IFA-100) and shown in Figure 1; the index difference, ∆n, between the core and cladding was 0.0016, mainly due to incorporation of Al in the fiber core. Note again that no Ge was added in the core. The concentrations of the Gd and Al ions were estimated to be 2.3 × 10^20^ ions/cm^3^ and 11.6 × 10^20^ ions/cm^3^, respectively. The core diameter, the outer diameter, and the cutoff wavelength of the Gd_2_O_3_ NPs-doped fiber were 7.3 µm, 125 µm, and 520 nm, respectively. Since the Faraday effect is considerably dependent on wavelength, i.e., it drastically decreases with increasing wavelength [30,31], the Faraday rotation measurement was carried out at visible wavelength, 650 nm. The refractive index profile was especially designed to have a numerical aperture of 0.0683 and a cutoff wavelength below 650 nm, over which single mode operates.

To verify the existence of Gd_2_O_3_ NPs in the core of the fiber, optical absorption spectrum of the Gd_2_O_3_ NPs-doped fiber was measured with a cut-back method using a few tens of centimeters of optical fiber length where the white light source (Ando Electric Co., Ltd., Tokyo, Japan AQ 4303B) was used for launching the input broadband light and output spectrum was measured with the optical spectrum analyzer (Ando Electric Co., Ltd., Tokyo, Japan, AQ 6315B).

### 3.4. Faraday Rotation Measurement

**Figure 2:**
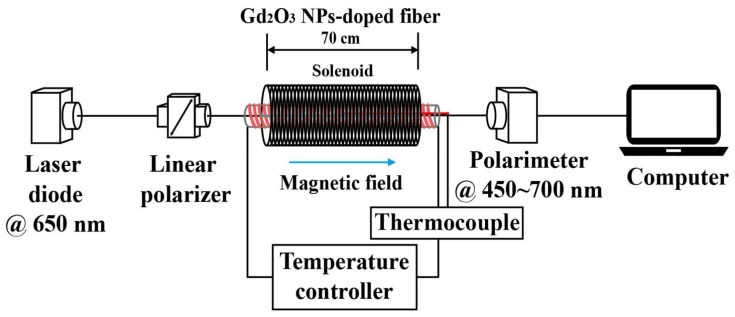
Experimental set-up to measure the temperature-dependent FRA of the Gd_2_O_3_ NPs-doped fiber under magnetic field induced by the DC solenoid.

To measure Faraday rotation angle (FRA) of the Gd_2_O_3_ NPs-doped fiber at 650 nm, a signal light from a superluminescent diode (SLD; Coset Inc., Gwangju, Korea, wavelength = 650 nm, power = 1 mW) was launched through a linear polarizer into the 70 cm long optical fiber that was under magnetic field generated by a DC solenoid with inner diameter of 76 mm (Walker scientific Inc., Worcester, MA, USA), as shown in Figure 2. In addition, to investigate temperature dependence of FRA, temperature variation was given by placing a glass tube heater inside a solenoid. The glass tube heater was made by winding a heating tape (nickel-chrome alloy wire) onto a silica glass tube with an inner diameter of 19 mm and a width of 3 mm. The fiber was aligned straight inside the glass tube under the magnetic field. The temperature was varied from 25 °C to 120 °C with a precision of 0.2 °C by an auto-tuning proportional-plus-integral-plus-derivative (PID) controller. FRA was measured by using a polarimeter (Thorlabs Inc., Newton, NJ, USA, PA510) and recorded in a Poincare sphere. The induced magnetic field obtained by the DC solenoid was varied up to 0.142 Tesla (T) at 25 °C–120 °C. The magnetic field distribution in the DC solenoid under an applied magnetic field of 0.142 T with varying temperatures from 25 °C to 120 °C was measured using the Gauss/Teslameter (Sypris Test and Measurement Inc., Orlando, FL, USA, Model 7010). No shielding effect of the magnetic field for each section along with the axial position in the DC solenoid at 25 °C–120 °C through the glass tube heater was found within uncertainty of about ±0.001 T. The Verdet constant, *V*, of the fiber was also estimated from the measured FRA, *θ*, by using the well-known Faraday equation [5,18,32]:

(4) θ=V∫Bdl=VBL,

in which *B* is the magnetic field in Tesla (T) and *L* is the length of the fiber under the influence of a magnetic field.

Finally, to verify the external vibration dependence of polarization state, an orientation of the polarization on the Poincare sphere was measured in various conditions of vibration with frequency from 20 Hz to 500 Hz and acceleration from 2 g to 20 g using the vibrator (Brüel & Kjær, Nærum, Denmark, LDS V830). The fiber of 70 cm length with one loop of 12 cm radius was attached onto the upper surface of the vibrator during the test. 

## 4. Results and Discussion

**Figure 3:**
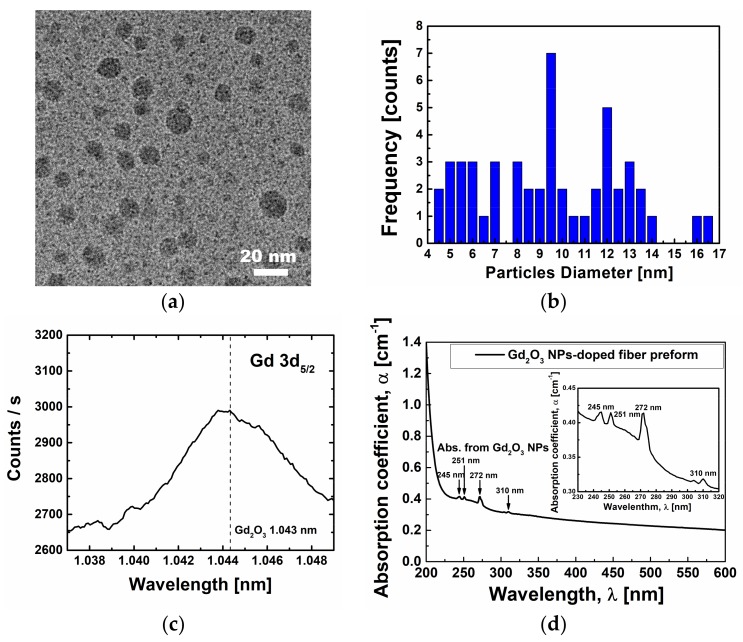
(**a**) TEM image; (**b**) size distribution; (**c**) XPS spectrum; and (**d**) UV-VIS spectrum of the Gd_2_O_3_ NPs-doped fiber preform.

To verify the existence of nano-particles in the core of the preform after its fabrication by using the MCVD process, direct TEM measurement or indirect absorption and kinetic energy measurements can be used. For the preform, the existence of Gd_2_O_3_ NPs in the core was confirmed by the TEM, which clearly showed the morphology and size distribution of Gd_2_O_3_ NPs. The particles were spherical and uniformly dispersed without agglomeration as shown in Figure 3a,b, respectively. The average diameter of Gd_2_O_3_ NPs was measured to be about 9.4 nm with the range of 4.5 nm–16.7 nm. Note that the added Al in silica glass played the role of modifying the glass network to be alumino-silicate glass and of increasing refractive index of the core. However, most of the doped Gd is believed to become oxidized to form Gd_2_O_3_ NPs, which was confirmed by the X-ray Photoelectron Spectroscopy (XPS) and the optical absorption. Furthermore, since Al and Gd dissolved in the doping solution were infiltrated into the porous silica glass layers during the doping process, the incorporated Gd_2_O_3_ NPs were uniformly dispersed due to random distribution of Al and Gd [33,34,35,36].

Figure 3c shows the XPS spectrum of the fiber preform to confirm the chemical state of Gd in the core. The binding energy was calibrated by centering the Si 2p peak at 103.3 eV. The Gd 3d_5/2_ peak located at 1.043 nm clearly indicates the existence of Gd_2_O_3_ NPs [37,38]. The UV-VIS absorption spectrum of the Gd_2_O_3_ NPs-doped fiber preform is shown in Figure 3d. Several distinct absorption peaks appeared in the UV region due to reorganization of the seven 4*f* electrons into various multiplets of Gd ions, attributed to the existence of the Gd_2_O_3_ NPs [10,39,40,41,42,43,44,45,46]. The absorption peaks appearing at 245 nm, 251 nm, 272 nm, and 310 nm can be assigned to ^8^S_7/2_ to ^6^D_3/2_, ^6^D_9/2_, ^6^I_5/2_, and ^6^P_7/2_ transitions in Gd_2_O_3_. Note that since Gd^3+^ ion has [Xe] 4*f*^7^ configuration and the ground state is ^8^S_7/2_, the absorption peak in Gd_2_O_3_ around 200 nm is attributed to the transition from ground ^8^S_7/2_ → ^6^G_J/2_ [44]. Moreover, other three lowest multiplets of ^6^P, ^6^I, and ^6^D above the ground ^8^S_7/2_ state remains. Energies of 14 states associated with ^6^P, ^6^I, and ^6^D multiplets are related to distinct absorptions from 240 nm to 320 nm [5,6,7,10,39,42,43,44]. In the case of Gd_2_O_3_ NPs, therefore, the distinct absorption peaks appeared over from 200 nm to 400 nm, corresponding to the transitions from the ground ^8^S_7/2_ to the excited ^6^P, ^6^I, and ^6^D multiplets [10].

**Figure 4:**
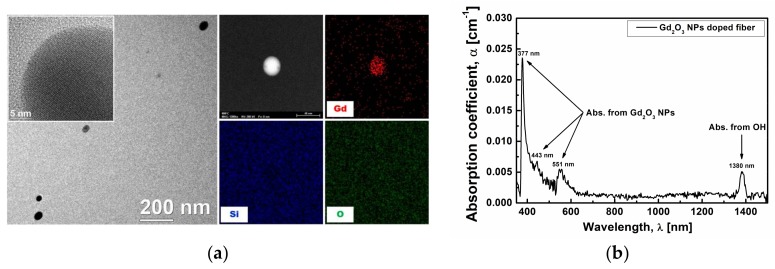
(**a**) TEM image with the EDS compositional maps for Gd, Si, and O and (**b**) absorption spectrum of the Gd_2_O_3_ NPs-doped fiber.

To confirm the retainment of Gd_2_O_3_ NPs in the fiber after the drawing the preform at high temperature (about 2150 °C), the existence of Gd_2_O_3_ NPs in the fiber core was also verified by the TEM and the absorption spectrum analysis as shown in Figure 4. Figure 4a shows the TEM image with the corresponding EDS compositional maps of Gd_2_O_3_ NPs in the optical fiber core. As seen in the TEM image, the average diameter of Gd_2_O_3_ NPs in the fiber core increased to 48 nm (size distribution: 35 nm–56 nm) from 9.4 nm in the preform. The increase in size with longitudinal growth of Gd_2_O_3_ NPs seems to be caused by the high temperature drawing process. The growth direction of Gd_2_O_3_ NPs is the same as the fiber drawing direction. In addition, the high resolution TEM image in the magnified inset image clearly shows that the embedded Gd_2_O_3_ NPs in the SiO_2_ glass matrix are crystalline. In addition, the EDS mapping clearly indicates that the nanoparticles consist of Gd. Note that the population density of the Gd_2_O_3_ NPs in the fiber decreased as compared with those in the preform because of the growth of Gd_2_O_3_ NPs and the elongation of the fiber during the drawing process. Also, as shown in Figure 4b, a distinct optical absorption was found to appear peaking at 377 nm, 443 nm, and 551 nm, and these absorptions are attributed to the existence of Gd_2_O_3_ NPs in the fiber core. The absorption peaks showed a superimposing tendency as compared with our previous work [26]. The absorption peaks at 377 nm, 443 nm, and 551 nm of the fiber seemed to red-shift from the corresponding peaks 251 nm, 272 nm, and 310 nm of the preform, respectively. This red-shift may be due to the increase in the size of Gd_2_O_3_ NPs in the fiber after the drawing process at high temperature. In the previous papers, the position of absorption peaks was found to shift towards long or short wavelength with the change in the size of the Gd_2_O_3_ NPs embedded in silica glass [7,10,26,42,43]. As shown in Figure 4b, the optical absorption peaks at 377 nm, 443 nm, and 551 nm can be attributed to ^8^S_7/2_ to ^6^D_9/2_, ^6^I_5/2_, and ^6^P_7/2_ transitions in Gd_2_O_3_ NPs, respectively. The intensities of absorption peaks at 377 nm, 443 nm, and 551 nm in Gd_2_O_3_ NPs of the fiber were changed to 0.02255 cm^−1^, 0.00189 cm^−1^, and 0.00427 cm^−1^ from 0.01709 cm^−1^, 0.36852 cm^−1^, and 0.00835 cm^−1^ at the corresponding peaks 251 nm, 272 nm, and 310 nm of the preform, respectively, after baseline correction. The absorption peak at 1380 nm was because of the OH impurities [47]. 

**Figure 5:**
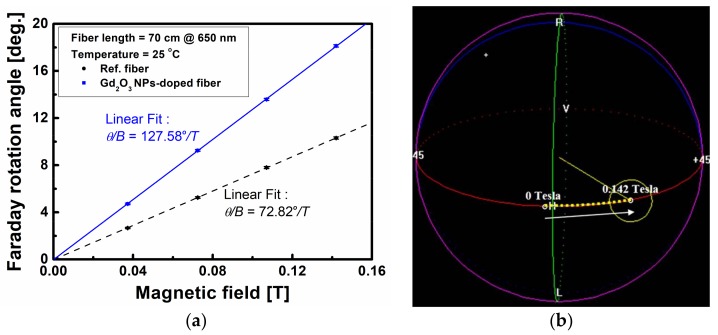
(**a**) Variation of the FRA with the increase of magnetic field at room temperature and (**b**) change in polarization states on a Poincare sphere of the Gd_2_O_3_ NPs-doped fiber.

FRA of the Gd_2_O_3_ NPs-doped fiber was measured at 650 nm using an SLD and a linear polarizer, based on the absorption spectrum and the availability of the fiber pigtailed optical components such that FRA viz. Verdet constant is known to increase with decreasing wavelength and especially approaching the absorption peak at 551 nm [30,31,48]. Figure 5a shows the measured FRA of the Gd_2_O_3_ NPs-doped fiber at 650 nm with the increase of the induced magnetic field at room temperature (25 °C). The change in polarization states on the Poincare sphere during the Faraday rotation measurement is indicated in Figure 5b. When the light signal from the SLD at 650 nm was passed through the linear polarizer, its polarization state (dashed yellow line) was nearly overlapped with the polarized state (red line) on the Poincare sphere. The rotation was found to be counterclockwise along the polarized state, and the rotation angle increased linearly with the increase of magnetic field. The FRA of the Gd_2_O_3_ NPs-doped fiber was about 18.13° at the magnetic field of 0.142 T. With the increment of applied magnetic field by varying DC current of the solenoid, the FRA of the fiber was found to increase linearly as shown in Figure 5a. The linear dependence was calculated to be *θ*/B = 127.58°/T, with the standard deviation of 0.38°/T. For a comparison, the FRA of the germano-silicate glass optical fiber but without Gd_2_O_3_ NPs as a reference fiber, which was fabricated using the same processing parameters during the MCVD and fiber drawing process to satisfy the cutoff wavelength below 650 nm, was also measured, and the linear dependence was calculated to be *θ/B* = 72.82°/T, with the standard deviation of 0.26°/T. The core diameter, the outer diameter, and the cutoff wavelength of the reference fiber were 8.7 µm, 125 µm, and 600 nm, respectively.

From the measured FRA and by using Equation (4), the Verdet constant of the present Gd_2_O_3_ NPs-doped fiber was estimated to be 3.18 rad/(T∙m), about 1.76 times larger than that of the reference fiber (1.81 rad/(T∙m)) at 650 nm. This increase can be explained by the fact that the 4*f*^n^ → 4*f*^n−1^5*d* transition of Gd ions becomes easier, because effect of compound ligand field on the inner 4*f* electron shell is very small due to shielding from the outer 5*s* and 5*p* electron shell [12]. Furthermore, a Zeeman splitting of atomic energy levels under an external magnetic field may result in magneto-optical rotation. For the Gd_2_O_3_ NPs-doped fiber, the *d* and *f* states of Gd ions contribute to the observed magneto-optic effect, because these states have not only a spin-obit interaction but also the exchange interaction between the electrons. In this regard, the large Verdet constant of the alumino-silicate glass optical fiber incorporated with Gd_2_O_3_ NPs can be attributed to the trivalent Gd^3+^ ions, which have seven unpaired free electrons of 4*f* electrons shell [4,5,12]. The magnetic response depends on the product of spin orbit interactions and the net spin polarization; *d* and *f* states in Gd^3+^ ions have the product term stronger than *p* and *s* conduction states. This results in the magneto-optic effect being dominated by electronic transition 4*f*^7^ → 4*f*^6^5*d*^1^, while 5*s* → 5*p* transition has negligible effect [12].

**Figure 6:**
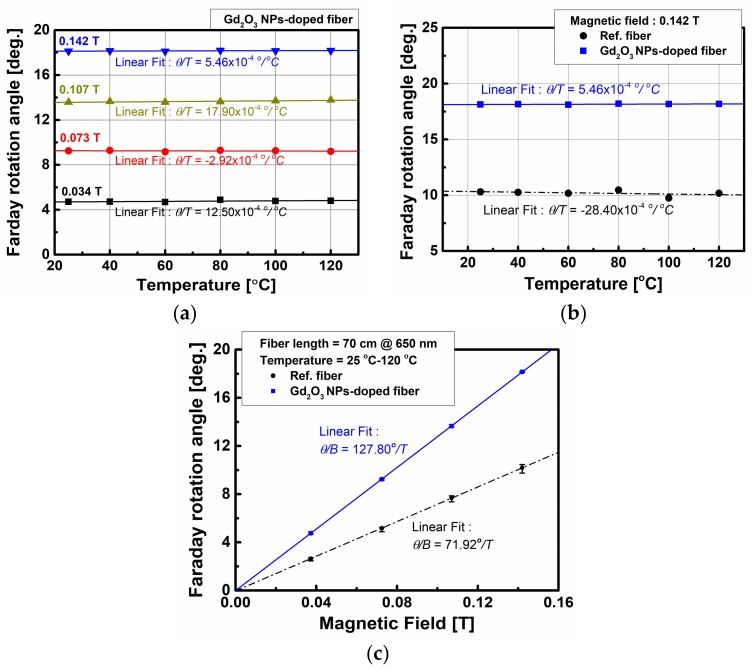
(**a**) Variation of the FRA of the Gd_2_O_3_ NPs-doped fiber with temperature under varied magnetic fields and comparison of the FRA of the Gd_2_O_3_ NPs-doped fiber and the reference fiber; (**b**) with temperature under 0.142 T; and (**c**) with magnetic field under varied temperature. The lines shown in the figures were obtained by the linear regression fitting.

Based on the measured results in Figure 5, the temperature dependence at 25 °C–120 °C of the FRA of the Gd_2_O_3_ NPs-doped fiber under induced magnetic field from 0.037 T to 0.142 T was investigated. Figure 6a shows the FRA with temperature at varied magnetic fields. With the increase of temperature, no appreciable change of FRA was found regardless of the magnetic field. Average temperature dependence, which is the slope of the line (*θ/T*), was 12.50 × 10^−4^, −2.92 × 10^−4^, 17.90 × 10^−4^, and 5.46 × 10^−4^ °/°C at 0.037, 0.073, 0.107, and 0.142 T, respectively. The present Gd_2_O_3_ NPs-doped fiber was almost independent of changes in temperature up to 120 °C (5.46 × 10^−4^ °/°C) and magnetic field up to 0.142 T. However, the temperature dependence of the reference fiber (−28.40 × 10^−4^ °/°C) was much larger (5.2 times) than that of the Gd_2_O_3_ NPs-doped fiber. 

The FRA was also plotted with magnetic field at various temperatures as shown in Figure 6b. With the increment of applied magnetic field by varying direct current (DC) at various constant temperatures, the FRA of the fiber was found to increase linearly with magnetic field. However, no appreciable deviation of the average FRA of the Gd_2_O_3_ NPs-doped fiber was found regardless of the temperature change, but that of the reference fiber increased. The average FRA and the deviation of the Gd_2_O_3_ NPs-doped fiber and the reference fiber were about 18.16° ± 0.048° and 10.19° ± 0.439° at 0.142 T at temperatures of 25 °C–120 °C, respectively. Thus, the resultant Verdet constant of the Gd_2_O_3_ NPs-doped fiber and the reference fiber at 0.142 T at 25 °C–120 °C were estimated to be 3.19 rad/(T·m) ± 0.01 rad/(T∙m) and 1.79 rad/(T·m) ± 0.08 rad/(T∙m), respectively. The experimental results on the temperature dependence of the Verdet constant are summarized in Table 1. 

**Figure 7:**
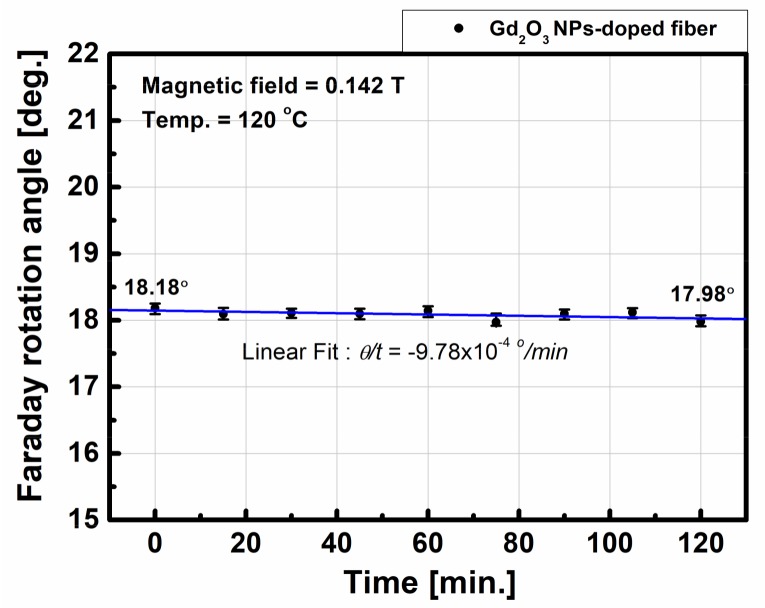
Variation of the FRA as function of the time under 0.142 T at 120 °C.

To examine the variation of FRA, if any, with time, the FRA of the Gd_2_O_3_ NPs-doped fiber was measured under 0.142 T at 120 °C with time at every 15 min for 120 min. The measured FRA change with time was −9.78 × 10^−4^ °/min. The change of the FRA after 120 min was 0.2° (Figure 7).

**Figure 8:**
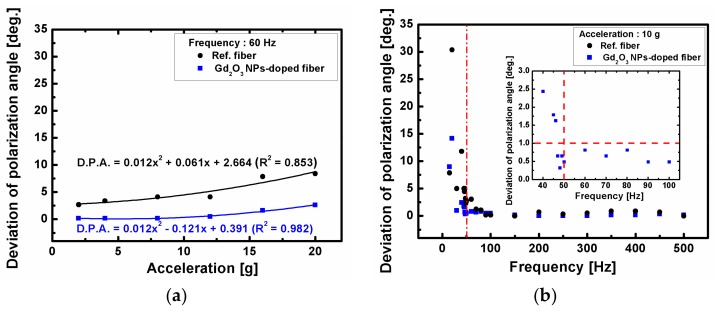
Vibration dependence of the Gd_2_O_3_ NPs-doped fiber and the reference fiber as a function of (**a**) acceleration under the frequency of 60 Hz and (**b**) frequency under the acceleration of 10 g. The inset in (**b**) shows the enlarged DPA of the Gd_2_O_3_ NPs-doped fiber from 45 Hz to 50 Hz with the interval of 1 Hz.

To examine the vibration dependence of FRA of the fibers, the FRA was measured under the vibration with acceleration from 2 g to 20 g with an interval 4 g under the frequency of 60 Hz. Figure 8 shows the vibration dependence of the Gd_2_O_3_ NPs-doped fiber and the reference fiber as a function of and frequency from 15 Hz to 500 Hz with an interval 50 Hz under the acceleration of 10 g. The deviation of polarization angle (DPA) of the Gd_2_O_3_ NPs-doped fiber and the reference fiber was found to increase from 0.163° to 2.604° and from 2.678° to 8.392°, respectively, with the increase of acceleration from 2 g to 20 g under the frequency of 60 Hz as shown in Figure 8a. However, both fibers showed large dependence on frequency (Figure 8b). The DPA increased sharply at low frequencies and soon dropped with the increase of the frequency near 50 Hz and then maintained (0.488°) up to 500 Hz under the acceleration of 10 g. The vibration dependence of the Gd_2_O_3_ NPs-doped fiber under varied acceleration and frequency was found to be smaller than that of the reference fiber. We found that the Gd_2_O_3_ NPs-doped fiber has minor DPA change under acceleration below 10 g and frequency above 50 Hz.

From the experimental results of the excellent insensitivity of temperature and vibration on the Faraday effect of the Gd_2_O_3_ NPs-doped fiber, the fiber incorporated with Gd_2_O_3_ NPs would be a good candidate for all-optical fiber magneto-optic devices, such as optical modulators, isolators, and current sensors. 

## 5. Conclusions

We fabricated the alumino-silicate glass optical fiber incorporated with Gd_2_O_3_ NPs by using the MCVD and the fiber drawing process, and investigated its magneto-optic sensing properties based on the Faraday effect. In particular, the temperature and vibration dependence of the Faraday effect of the fiber was measured and discussed.

Uniformly embedded Gd_2_O_3_ NPs with average diameter of 9.4 nm were identified to form in the core of the fiber preform. The average diameter of Gd_2_O_3_ NPs in the fiber core was found to increase from 9.4 nm to 48 nm due to the recrystallization and growth of Gd_2_O_3_ NPs during the high temperature fiber drawing process. The core diameter, the outer diameter, the cutoff wavelength, and the numerical aperture of the fabricated optical fiber were 7.3 µm, 125 µm, 520 nm, and 0.0683, respectively. The Gd 3d_5/2_ peak located at 1.043 nm and the absorption peaks appearing at 377 nm, 443 nm, and 551 nm are attributed to the Gd_2_O_3_ NPs existed in the fiber core. The FRA measured at 650 nm was found to increase linearly with the magnetic field, and the linear dependence (*θ/B*) was 127.58°/T at 25 °C. The Verdet constant was estimated to be 3.18 rad/(T∙m), about 1.76 times larger than that of the reference fiber, 1.81 rad/(T∙m). 

As for the temperature dependence of the FRA of the Gd_2_O_3_ NPs-doped fiber, no appreciable change of FRA (*θ/T* = 5.46 × 10^−4^ °/°C at 0.142 T) was found with the increase of temperature from 25 °C to 120 °C. The variation of the FRA with time at 0.142 T and 120 °C was negligibly small (−9.78 × 10^−4^ °/min). The average Verdet constant of the Gd_2_O_3_ NPs-doped fiber at 0.142 T at 25 °C–120 °C was estimated 3.19 rad/(T·m) ± 0.01 rad/(T∙m). The variation of the FRA under the mechanical vibration with the acceleration below 10 g and the frequency above 50 Hz was within 0.5°.

## Figures and Tables

**Table 1:** Verdet constants of the Gd_2_O_3_ NPs-doped fiber upon applying the magnetic fields at various constant temperatures.

		Verdet Constant (rad/(T·m))				
		at 0.037 T	at 0.073 T	at 0.107 T	at 0.142 T	Average
Temperature (°C)	25	3.15	3.18	3.16	3.18	3.17
	40	3.16	3.19	3.18	3.19	3.18
	60	3.13	3.15	3.16	3.18	3.16
	80	3.26	3.20	3.18	3.19	3.21
	100	3.19	3.18	3.19	3.19	3.19
	120	3.20	3.16	3.21	3.19	3.19
